# Identification and Application of a Panel of Constitutive Promoters for Gene Overexpression in *Staphylococcus aureus*

**DOI:** 10.3389/fmicb.2022.818307

**Published:** 2022-02-28

**Authors:** Qiang Liu, Daiyu Li, Ning Wang, Gang Guo, Yun Shi, Quanming Zou, Xiaokai Zhang

**Affiliations:** ^1^West China Biopharmaceutical Research Institute, West China Hospital, Sichuan University, Chengdu, China; ^2^National Engineering Research Center of Immunological Products, Department of Microbiology and Biochemical Pharmacy, College of Pharmacy, Third Military Medical University, Chongqing, China

**Keywords:** *S. aureus*, highly expressed gene, constitutive promoter, gene expression, PurR, catalase

## Abstract

*Staphylococcus aureus* is a leading pathogen that is currently the most common cause of infection in hospitalized patients. An in-depth genetic analysis of *S. aureus* virulence genes contributing to pathogenesis is needed to develop novel antimicrobial therapies. However, tools for genetic manipulation in *S. aureus* are limited, particularly those for gene expression. Here, 38 highly expressed genes were identified in *S. aureus* USA300_FPR3757 via RNA-seq. Promoter regions from 30 of these genes were successfully cloned, of which 20 promoters exhibited a wide range of activity. By utilizing these active promoters, 20 *S. aureus*-*Escherichia coli* shuttle vectors were constructed and evaluated by expressing an *egfp* reporter gene. Expression of the *egfp* gene under the control of different promoters was confirmed and quantified by Western blotting and qPCR, which suggested that the activity of these promoters varied from 18 to 650% of the activity of P*_*sarA*_*, a widely used promoter for gene expression. In addition, our constructed vectors were verified to be highly compatible with gene expression in different *S. aureus* strains. Furthermore, these vectors were evaluated and used to overexpress two endogenous proteins in *S. aureus*, namely, catalase and the transcriptional repressor of purine biosynthesis (PurR). Meanwhile, the physiological functions and phenotypes of overexpressed PurR and catalase in *S. aureus* were validated. Altogether, this evidence indicates that our constructed vectors provide a wide range of promoter activity on gene expression in *S. aureus*. This set of vectors carrying different constitutive promoters developed here will provide a powerful tool for the direct analysis of target gene function in staphylococcal cells.

## Introduction

*Staphylococcus aureus* is a major Gram-positive opportunistic pathogen causing both community-acquired and hospital-acquired infections in humans. The success of *S. aureus* as a leading pathogen is undoubtedly attributed to its severe antibiotic resistance as well as the extensive repertoire of virulence factors that enable it to evade the host immune system (Turner et al., 2019). An increased understanding of the pathogenicity and antibiotic resistance mechanisms of *S. aureus* is necessary to identify potential targets for the development of novel antimicrobial therapies. Therefore, an in-depth genetic analysis of *S. aureus* virulence genes contributing to pathogenesis is highly desirable.

Characterization of genes of unknown function in *S. aureus* has traditionally been conducted via gene knockout followed by complementation, as well as overexpression of the gene product using an inducible or constitutive promoter. Several shuttle vectors, including pBT2, pKOR1, pMAD, and a range of pRLY2, are currently available for gene disruption in *S. aureus* via double homologous recombination (Bruckner, 1997; Arnaud et al., 2004; Bae and Schneewind, 2006; Redder and Linder, 2012). However, the system for gene expression is relatively limited, largely due to the lack of well-characterized functional promoters in *S. aureus*.

Several inducible promoters have been applied in *S. aureus*. These include the *Bacillus megaterium*-derived xylose inducible promoter (Wieland et al., 1995; Kim et al., 1996; Yepes et al., 2014; Shang et al., 2019), the IPTG (isopropyl-beta-D-thiogalactopyranoside)-inducible promoter from the pSpac-lacI system (Gardete et al., 2004; Pereira et al., 2007; Liew et al., 2011), and the tetracycline-regulated hybrid promoter P*_*xyl–tetO*_* (Bateman et al., 2001; Corrigan and Foster, 2009). Although these inducible promoters have been used for gene expression in *S. aureus*, some limitations have yet to be resolved. For example, the pSpac/lacI system is not readily adaptable to *S. aureus* due to its high basal promoter activity (Bateman et al., 2001; Liew et al., 2011). In addition, induction with IPTG renders this system less attractive in an animal model system. The xylose-inducible promoter system is repressible by glucose (Wieland et al., 1995), which also prohibits its use in *in vivo* studies, as glucose is a common constituent inside mammalian cells. The tetracycline-inducible promoter system is currently the most functional inducible system for gene expression in *S. aureus* (Bateman et al., 2001; Harrison et al., 2019; Goncheva et al., 2020; Kuiack et al., 2020). However, the inducers such as tetracycline or anhydrotetracycline required in this system, in our experience, affect the normal metabolism of or are even toxic to *S. aureus*, which results in undesirable phenotypes.

Given the deficiencies of inducible systems, constitutive promoter systems, with no need for inducer substances, possess the advantage of making them ideally suitable for functional investigation of specific genes in *S. aureus*, particularly in an *in vivo* infection model. However, only a few constitutive promoters are currently available for gene expression in *S. aureus*. These include the global regulator *sarA* gene promoter and the type 1 capsule gene promoter P*cap*. The *sarA* gene consists of three promoters upstream of the *sarA* gene, the proximal P1 and distal P3 and P2 promoters (Bayer et al., 1996; Cheung and Manna, 2005). *sarA*-P1 has the strongest promoter activity and is thus widely employed for expressing various fluorescent reporter genes constitutively in *S. aureus* (Cheung et al., 1998; Malone et al., 2009; de Jong et al., 2017), which makes *S. aureus* able to be tracked and monitored both *in vitro* and *in vivo* (Boero et al., 2021; Yu et al., 2021). Other than the promoter *sarA*-P1, the serotype 1 capsule gene promoter P*cap* has been characterized to have constitutively strong activity in *S. aureus* (Ouyang and Lee, 1997; Schwendener and Perreten, 2015). Using the P*cap* promoter, an *Escherichia coli*–*S. aureus* shuttle vector pBUS1-P*cap*-HC has been constructed for gene expression in both *E. coli* and *S. aureus* (Schwendener and Perreten, 2015).

Although few constitutive promoters are available for gene expression in *S. aureus*, it is still limited for expressing a gene constitutively at a desired level. For example, when it is needed for the expression of genes with potential toxic effects, such as some transcriptional repressors, the transcriptional level of the target gene should be controlled at an appropriate level. Protein overexpression due to a strong constitutive promoter may be lethal to cells. However, insufficient protein expression and the control of a weak promoter might lead to insignificant phenotypes, which hinder the real function of the gene to be uncovered. Therefore, constitutive promoters with a wide range of activity provide the possibility of gene expression at a desirable level and may be utilized in investigating various virulence genes in *S. aureus*.

In this study, we identified a set of highly expressed genes during different growth phases of *S. aureus*. The promoters of these highly expressed genes were cloned and evaluated in terms of their activity. Based on the active promoters, a set of vectors was constructed for gene overexpression. The promoter strength of each vector was quantified by expressing an *egfp* reporter gene. Additionally, the universality of these vectors was evaluated in different *S. aureus* strains. Furthermore, two endogenous proteins, namely, PurR and catalase, were successfully expressed using our constructed vectors. Moreover, phenotypes of *S. aureus* overexpressing PurR or catalase were checked and confirmed. We believe that the set of vectors we developed may be utilized in investigating the contribution of virulence genes responsible for the pathogenesis of *S. aureus*.

## Results

### Screening of Highly Expressed Genes in *S. aureus* USA300_FPR3757 via RNA-Seq

**FIGURE 1:**
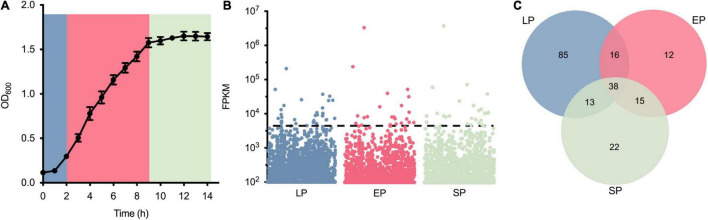
Identification of highly expressed genes from *Staphylococcus aureus* USA300_FPR3757. **(A)** Growth curve of *S. aureus* USA300_FPR3757 growing at 37°C in TSB medium. **(B)** Normalized expression level (FPKM) of genes in the LP, EP, and SP growth phases. Each point represents a gene, and the dashed line indicates the cutoff value of FPKM 1,500. **(C)** Venn diagram representing the number of shared highly expressed genes (FPK ≥ 1,500) in the LP, EP, and SP growth phases.

Thirty-eight genes highly expressed in three different growth phases were screened via RNA-seq (Figure 1). To identify the highly expressed genes in different growth phases, the growth curve of *S. aureus* USA300_FPR3757 in TSB medium was first drawn (Figure 1A). Bacterial cells from different time points (2, 6, or 10 h) after inoculation, corresponding to the lag phase (LP), exponential phase (EP), and stationary phase (SP), were collected for RNA extraction and subsequently submitted for RNA-seq. The transcriptome dataset, including read counts and fragments per kilobase million (FPKM) of each gene from LP, EP, and SP, is listed in Supplementary Table 1. The expression levels of all 6,030 genes during LP, EP, and SP were ranked from the most highly expressed to the least expressed according to their FPKM values (Figure 1B). Genes with FPKM values ≥ 1,500 were defined as highly expressed, and there were 152, 81, and 88 highly expressed genes in the LP, EP, and SP growth phases, respectively (Figures 1B,C). Among these genes, 38 genes were all highly expressed in all three growth phases (Figure 1C).

### Cloning of the Promoter Regions of Identified Highly Expressed Genes

Since the exact -35 and -10 sequences of these promoters were unknown, the gap non-coding sequence between the highly expressed gene and its upstream gene was defined as the potential promoter sequence. Among these 38 highly expressed genes, there were eight genes with very short promoter regions or no promoter region, making them difficult or impossible to clone. Therefore, the promoter regions of the other 30 genes were selected for cloning and evaluation (Supplementary Table 3). If the promoter sequence was longer than 450 bp, then only 450 bp was chosen as a promoter sequence. In addition, the commonly used constitutive promoter of the transcriptional regulator gene *sarA* P*_*sarA*_* (P31) was also cloned as a positive control (Bayer et al., 1996; Cheung and Manna, 2005). The length of the selected promoters ranged from 120 to 434 bp, and each promoter was named the gene locus of its corresponding gene. Information on the cloned promoters, including their length, coding products of their corresponding genes, and the transcriptional level (FPKM value) of the selected gene, is shown in Table 1. In short, among these 38 highly expressed genes, the promoter regions of 30 genes were successfully cloned (Table 1).

**TABLE 1:** Cloned promoter regions of highly expressed genes of *S. aureus* USA300_FPR3757.

No.	Name of promoter	Gene locus	Promoter length (bp)	CDS products	Transcriptional level (FPKM)		
					LP	EP	SP
P1	P*_10930_*	SAUSA300_RS10930	235	Delta-lysin family phenol-soluble modulin	2.1E+05	2.7E+06	3.0E+06
P2	P*_13425_*	SAUSA300_RS13425	434	Hypothetical protein	5.1E+04	2.0E+05	5.2E+04
P3	P*_01490_*	SAUSA300_RS01490	247	Virulence factor EsxA	2.4E+04	4.7E+04	3.4E+04
P4	P*_05790_*	SAUSA300_RS05790	363	Beta-class phenol-soluble modulin	2.8E+03	3.6E+04	6.3E+04
P5	P*_12390_*	SAUSA300_RS12390	297	HTH-type transcriptional regulator SarR	2.4E+03	8.1E+03	9.5E+03
P6	P*_04840_*	SAUSA300_RS04840	294	Transcriptional regulator Spx	5.7E+03	7.6E+03	6.9E+03
P7	P*_04400_*	SAUSA300_RS04400	350	CsbD family protein	8.9E+03	7.4E+03	1.5E+04
P8	P*_10935_*	SAUSA300_RS10935	235	Accessory gene regulator AgrB	3.1E+03	7.2E+03	8.0E+03
P9	P*_04190_*	SAUSA300_RS04190	356	Cold-shock protein	5.2E+03	6.7E+03	2.1E+03
P10	P*_00165_*	SAUSA300_RS00165	150	PBP2a family beta-lactam-resistant peptidoglycan transpeptidase MecA	7.3E+03	5.2E+03	3.5E+03
P11	P*_02850_*	SAUSA300_RS02850	216	Elongation factor Tu	1.5E+04	5.0E+03	5.9E+03
P12	P*_03960_*	SAUSA300_RS03960	120	Ribosomal subunit interface protein	9.2E+03	4.7E+03	6.3E+03
P13	P*_05175_*	SAUSA300_RS05175	301	Quinol oxidase subunit 2	2.9E+03	4.5E+03	2.7E+03
P14	P*_11445_*	SAUSA300_RS11445	217	Fructose-bisphosphate aldolase	1.8E+04	4.4E+03	2.3E+03
P15	P*_12155_*	SAUSA300_RS12155	356	30S ribosomal protein S10	1.3E+04	4.1E+03	2.8E+03
P16	P*_12125_*	SAUSA300_RS12125	200	50S ribosomal protein L22	9.5E+03	3.0E+03	3.2E+03
P17	P*_11990_*	SAUSA300_RS11990	239	50S ribosomal protein L13	7.9E+03	2.9E+03	1.6E+03
P18	P*_11805_*	SAUSA300_RS11805	120	Asp23/Gls24 family envelope stress response protein	3.3E+03	2.9E+03	4.0E+03
P19	P*_11815_*	SAUSA300_RS11815	174	Alkaline shock response membrane anchor protein AmaP	3.2E+03	2.7E+03	3.8E+03
P20	P*_08825_*	SAUSA300_RS08825	216	Glutamyl-tRNA reductase	1.6E+03	2.4E+03	2.0E+03
P21	P*_02795_*	SAUSA300_RS02795	180	50S ribosomal protein L11	1.0E+04	2.0E+03	2.7E+03
P22	P*_12040_*	SAUSA300_RS12040	192	Translation initiation factor IF-1	6.1E+03	2.0E+03	1.9E+03
P23	P*_02845_*	SAUSA300_RS02845	122	Elongation factor G	4.2E+03	2.0E+03	1.8E+03
P24	P*_02805_*	SAUSA300_RS02805	271	50S ribosomal protein L10	9.6E+03	1.9E+03	2.2E+03
P25	P*_08620_*	SAUSA300_RS08620	150	CsbD family protein	5.1E+03	1.8E+03	2.9E+03
P26	P*_02835_*	SAUSA300_RS02835	150	30S ribosomal protein S12	4.0E+03	1.8E+03	1.5E+03
P27	P*_12130_*	SAUSA300_RS12130	200	30S ribosomal protein S19	5.8E+03	1.7E+03	1.8E+03
P28	P*_12070_*	SAUSA300_RS12070	150	50S ribosomal protein L18	5.4E+03	1.7E+03	1.8E+03
P29	P*_12030_*	SAUSA300_RS12030	167	30S ribosomal protein S13	5.6E+03	1.6E+03	1.5E+03
P30	P*_02545_*	SAUSA300_RS02545	150	RidA family protein	3.0E+03	1.6E+03	1.8E+03
P31	P*_sarA_*	SAUSA300_RS03250	264	Transcriptional regulator SarA	7.2E+02	9.2E+02	1.4E+03

### Characterization of the Cloned Promoters by Beta-Galactosidase Assay

**FIGURE 2:**
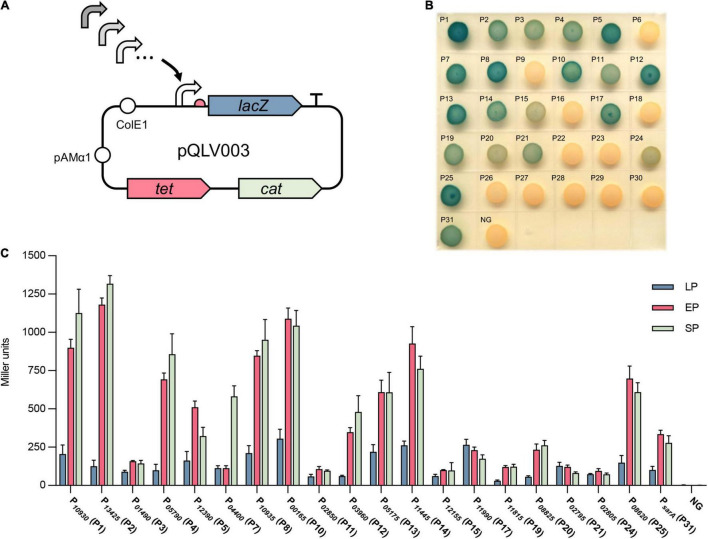
Evaluation of the activity of selected promoters by beta-galactosidase assay. **(A)** Selected active promoters were cloned into the promoter-probe vector pQLV003 carrying a *lacZ* reporter gene. The pQLV003 vector contains the *Escherichia coli* origin ColE1, pAMα1 replicon for propagation in Gram-positive bacteria, the selectable marker tetracycline (tet) in *E. coli* and chloramphenicol (*cat*) in *S. aureus.* **(B)** The vector pQLV003 carrying each selected promoter in front of the *lacZ* gene was transformed into *S. aureus* USA300. The resulting strains were grown on TSB *X*-gal plates at 37°C for 24 h. **(C)** The beta-galactosidase activity of the cell lysate of *S. aureus* USA300 transformed with each promoter_*lacZ* plasmid at lag phase (LP), exponential phase (EP), and stationary phase (SP). NG represents the negative control in which the *egfp* gene was driven by no promoter.

To check the activity of the selected promoters, the promoter-probe vector pQLV1003 carrying the *lacZ* reporter gene was constructed as described in the methods and materials (Table 2). All the selected promoters were amplified and cloned into pQLV1003 upstream of a *lacZ* reporter gene (Figure 2A). The generated plasmids were transformed into *S. aureus* USA300, and the activity of each cloned promoter was evaluated by measuring the beta-galactosidase activity of each transformed strain.

**TABLE 2:** Bacterial strains and plasmids used in this study.

Strain	Characteristics	Source
DH5α	K-12 strain; *recA1 endA1 hsdR17* (r_K_^–^ m_K_^+^)	Laboratory strain
RN4220	A restriction minus derivative of *S. aureus* strain 8325-4	Laboratory strain
*S. aureus* ATCC10832	Surface protein A-negative *S. aureus* Wood 46 (ATCC10832)	Laboratory strain
*S. aureus* USA300_FPR3757	USA300 LAC; hypervirulent community-associated MRSA, cured of antibiotic resistance plasmid	Laboratory strain
*S. aureus* NCTC_8325	Reference strain	Laboratory strain
*S. aureus* Newman	*S. aureus* strains expressing coagulase	Baba et al., 2008
*S. aureus* MW2	A typical community-acquired strain of MRSA	Laboratory strain
**Plasmid**		
pTH100	*egfp* reporter gene plasmid	de Jong et al., 2017
pBUS1-HC	*S. aureus*–*E. coli* shuttle vector; pAMα1 minimum replicon; *sso oriL* ColE1; MCS pBluescript II SK (Stratagene); (*rrnB* T1)5, *tet*(L)	Schwendener and Perreten, 2015
pBUS1-P_cap_-HC	*S. aureus*–*E. coli* shuttle vector; pAMα1 minimum replicon; *sso oriL* ColE1; P*_*cap*_*-MCS-*rgs-his_6_*, (*rrnB* T1)5; *tet*(L)	Schwendener and Perreten, 2015
pQLV1001	pBUS1-HC derived vector pBUS1-HC_*cat*; a chloramphenicol resistance cassette (*cat*) was inserted into pBUS1-HC at *Bgl*II site	This study
pQLV1002	pBUS1-P*_*cap*_*-HC derived vector pBUS1-P*_*cap*_*-HC_*cat*; a chloramphenicol resistance cassette (*cat*) was inserted into pBUS1-HC at *Bgl*II site	This study
pQLV1003	pQLV1001-derived plasmid pBUS1_HC_*cat*_*lacZ*; promoterless *lacZ* reporter vector (*rfp*)_ *lacZ* gene fragment was inserted into pQLV1001 at *Hin*dIII and *Bam*HI sites	This study
pQLV1010	pQLV1002-derived expression vector carrying the P*_10930_* (P1) promoter	This study
pQLV1011	pQLV1002-derived expression vector carrying the P*_13425_* (P2) promoter	This study
pQLV1012	pQLV1002-derived expression vector carrying the P*_01490_* (P3) promoter	This study
pQLV1013	pQLV1002-derived expression vector carrying the P*_05790_* (P4) promoter	This study
pQLV1014	pQLV1002-derived expression vector carrying the P*_12390_* (P5) promoter	This study
pQLV1015	pQLV1002-derived expression vector carrying the P*_04400_* (P7) promoter	This study
pQLV1016	pQLV1002-derived expression vector carrying the P*_10935_* (P8) promoter	This study
pQLV1017	pQLV1002-derived expression vector carrying the P*_00165_* (P10) promoter	This study
pQLV1018	pQLV1002-derived expression vector carrying the P*_02850_* (P11) promoter	This study
pQLV1019	pQLV1002-derived expression vector carrying the P*_03960_* (P12) promoter	This study
pQLV1020	pQLV1002-derived expression vector carrying the P*_05175_* (P13) promoter	This study
pQLV1021	pQLV1002-derived expression vector carrying the P*_11445_* (P14) promoter	This study
pQLV1022	pQLV1002-derived expression vector carrying the P*_12155_* (P15) promoter	This study
pQLV1023	pQLV1002-derived expression vector carrying the P*_11990_* (P17) promoter	This study
pQLV1024	pQLV1002-derived expression vector carrying the P*_11815_* (P19) promoter	This study
pQLV1025	pQLV1002-derived expression vector carrying the P*_08825_* (P20) promoter	This study
pQLV1026	pQLV1002-derived expression vector carrying the P*_02795_* (P21) promoter	This study
pQLV1027	pQLV1002-derived expression vector carrying the P*_02805_* (P24) promoter	This study
pQLV1028	pQLV1002-derived expression vector carrying the P*_08620_* (P25) promoter	This study
pQLV1029	pQLV1002-derived expression vector carrying the P*_sarA_* (P31) promoter	This study

First, the beta-galactosidase activity of the transformed strains was tested on a TSB X-gal plate. The results showed that 20 strains exhibited blue colonies on the plate (Figure 2B), with the corresponding 20 promoters (P1, P2, P3, P4, P5, P7, P8, P10, P11, P12, P13, P14, P15, P17, P19, P20, P21, P24, and P25) and the positive control P*_*sarA*_* (P31). Subsequently, the strength of these 20 verified active promoters was quantified by measuring the beta-galactosidase activity of their corresponding bacterial cell lysates (Figure 2C). Overall, the activity of most promoters in EP (6 h) and SP (10 h) was significantly stronger than that in early growing phase LP (2 h), except P3, P11, P15, P17, P19, P21, and P24, which exhibited a similar activity in all growing phases. The activity of most promoters exhibited no significant difference (*p*-value ≥ 0.05) in EP (6 h) and SP (10 h) with the exception of P1, P2, P4, P5, P7, and P12, among which P1, P2, P4, P7, and P12 had stronger activity in SP and P5 had stronger activity in EP. Compared with the commonly used promoter P*_*sarA*_* (P31), 11 promoters displayed stronger activity. Overall, the activity of the cloned 20 promoters varied from 25 to 500% of the activity of P*_*sarA*_*. Vector pQLV1003 without a promoter served as a negative control (NG), which exhibited no beta-galactosidase activity. In summary, the activity of these 30 selected promoters was evaluated in the native host, and 20 promoters showed a wide range of activity.

### Construction of Overexpression Vectors Based on the Selected Promoters

**FIGURE 3:**
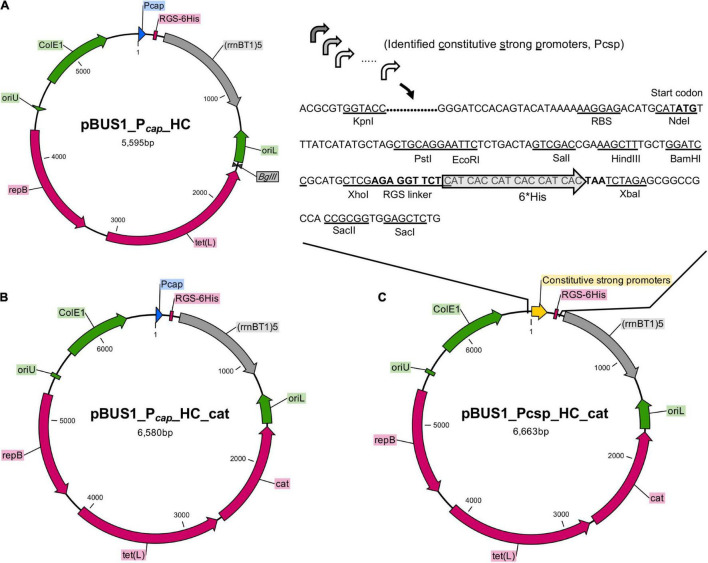
Construction of overexpression vectors by using the active promoters. **(A)** The backbone vector pBUS1_P*_*cap*_*_HC was used for the development of the set of overexpression vectors. The elements in the backbone vector pBUS1_P*_*cap*_*_HC include the P*_*cap*_* promoter, *E. coli* origin ColE1, the terminator sequence (rrnB T1)5 and the selectable marker tetracycline *tet* (L). The elements required for rolling-circle replication are indicated: the replication initiator protein gene (*repB*), the double-strand origin (*oriU*), and the single-strand origin (*oriL*). **(B)** The chloramphenicol cassette (*cat*) was inserted into pBUS1_P*_*cap*_*_HC at the *Bgl*II site to generate the plasmid pBUS1_P*_*cap*_*_HC_*cat*. **(C)** The DNA fragment containing each individual selected promoter sequence and a ribosomal-binding site (RBS) was cloned into pBUS1_P*_*cap*_*_HC_*cat* at the *Kpn*I and *Nde*I sites, which replaced the original P*_*cap*_* promoter sequence, to generate a set of expression vectors carrying different constitutive and active promoters.

Using the identified 20 active promoters, 20 vectors derived from pBUS1_P*_*cap*_*_HC were constructed for constitutive protein expression in *S. aureus*. pBUS1_P*_*cap*_*_HC is a high-copy *S. aureus*-*E. coli* shuttle vector used for protein overexpression in *S. aureus* using the promoter of type 1 capsule gene 1A (P*_*cap*_*) (Schwendener and Perreten, 2015) (Figure 3A). It contains the tetracycline marker for plasmid selection in both *E. coli* and *S. aureus*. Because some clinical *S. aureus* strains, such as *S. aureus* USA300 and *S. aureus* MW2, are resistant to tetracycline, an additional selection marker, the chloramphenicol cassette (*cat*), was amplified from pBT2 (Bruckner, 1997) and introduced into pBUS1_P*_*cap*_*_HC at the *Bgl*II site, generating the vector pBUS1_P*_*cap*_*_HC_*cat* (pQLV1002) (Figure 3B).

To generate the overexpression vectors by applying our identified constitutive promoters, the P*_*cap*_* promoter sequence from the pBUS1_P*_*cap*_*_HC_*cat* plasmid was replaced with a new DNA fragment, which contained an individual selected promoter sequence and a typical ribosomal-binding site (RBS) (Figures 3B,C). This generated a set of vectors harboring the identified strong constitutive promoter individually (Figure 3C). Downstream of the promoter, the multiple cloning site (MCS) and the RGS-6 × His coding sequence were the same as those in the original vector pBUS1_P*_*cap*_*_HC_*cat*, which enables the expression of the target protein with a C-terminal 6 × His tag (Figure 3C). Briefly, 20 vectors carrying different constitutive promoters were developed for gene expression and the production of tagged fusion proteins.

### Evaluation and Quantification of the Constructed Vectors by Expressing an *egfp* Reporter Gene

**FIGURE 4:**
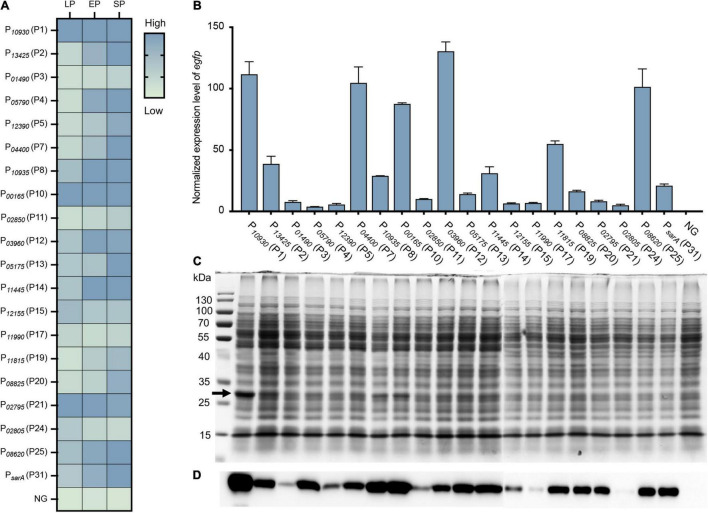
Evaluation and quantification of the strength of constructed vectors by expressing an *egfp* reporter gene. **(A)** Fluorescence-based promoter activity assay. The *egfp* reporter gene was cloned into each vector under the control of different promoters. The resulting strain was transformed into *S. aureus* USA300, and the fluorescence intensity and OD_600_ value of each strain were measured by a Bioreader at the LP, EP, and SP growth phases. The value of the ratio fluorescence intensity/OD_600_ ratio was calculated. **(B)** Additionally, the transcriptional level of the *egfp* gene under different promoters was assessed by RT-qPCR. The expression level was normalized to the internal control *sigA* gene. Data shown are the mean ± SD of three experiments. **(C)** The production of recombinant eGFPs was checked by SDS-PAGE and **(D)** Western blotting using an anti-6 × His tag antibody. NG represents the negative control wherein the *egfp* gene was driven by no promoter.

To evaluate the effect and efficiency of the constructed expression vectors on gene expression, an e*gfp* reporter gene was cloned into the MCS of each vector at the *Nde*I and *Xho*I sites, which allowed the e*gfp* gene to be expressed with an RGS_6 × His tag under the control of different promoters. The verified plasmid was transformed into *S. aureus* USA300, and the fluorescence and OD_600_ value of each resulting strain were measured in a bioreader. Promoter activity was analyzed by calculating the ratio of RLU/OD_600_ (Supplementary Table 4), and a heatmap based on the RLU/OD_600_ value in different growth phases was generated (Figure 4A). All tested promoters were active in LP, EP, or SP compared to the negative control, in which the *egfp* gene was driven by no promoter (Figure 4A).

To further quantify the strength of these vectors on gene expression, the transcriptional level of the *egfp* gene in each vector was quantified by RT-qPCR. The *egfp* gene was overexpressed in all vectors compared to the negative control (NG) (Figure 4B), which further confirmed the effect of our constructed vectors on gene expression. When compared to the commonly used promoter P*_*sarA*_* (P31), nine promoters (P1, P2, P7, P8, P10, P12, P14, P19, and P25) were stronger than P*_*sarA*_* (P31), while 10 promoters were weaker than P*_*sarA*_* (P31) (Figure 4B). Overall, the activity of our selected promoters in the overexpression vectors varied from 18 to 650% of the activity of P*_*sarA*_* (P31) (Figure 4B).

Additionally, the expression of the eGFP was confirmed by SDS-PAGE and western blotting. Overproduction of the recombinant eGFPs (28 kDa) from some strong promoters, such as P*_10930_* (P1), P*_10935_* (P8), and P*_00165_* (P10), was clearly visible on the SDS-PAGE gel (Figure 4C). Owing to the eGFP being expressed with a 6 × His tag at its C-terminal, the expression of the eGFP was further confirmed by western blotting using an anti-6 × His tag antibody. The recombinant eGFP was detected in all strains at various levels (Figure 4D), most of which corresponded to their transcriptional level (Figures 4C,D). Collectively, all the overexpression vectors constructed here efficiently expressed their target genes. More importantly, this set of vectors displayed a wide range of activity in their effects on gene expression.

### Evaluation of the Gene Overexpression Vectors in Different *S. aureus* Strains

**FIGURE 5:**
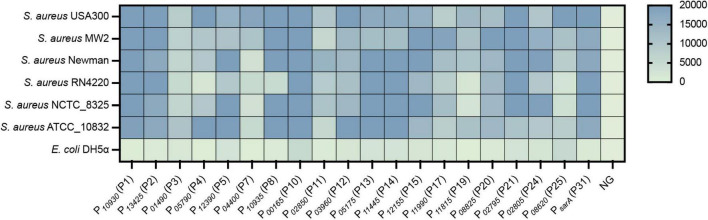
The constructed vectors were evaluated in different *S. aureus* strains. The *egfp* reporter gene was cloned into each vector under the control of different promoters. The resulting plasmid was transformed into different *S. aureus* strains, and the fluorescence intensity and OD_600_ value of each strain were measured by a Bioreader at the exponential phase. The fluorescence intensity/OD_600_ ratio was calculated. NG represents the negative control in which the *egfp* gene was driven by no promoter.

To evaluate the universality of the constructed expression vectors, an *egfp* gene was cloned into each vector and transformed into different *S. aureus* strains, which included *S. aureus* USA300, *S. aureus* MW2, *S. aureus* Newman, *S. aureus* RN4220, *S. aureus* NCTC_8325, and *S. aureus* ATCC 10832. As shown, the eGFP was successfully overexpressed in all vectors compared to the negative control, in which the *egfp* gene was regulated without a promoter (Figure 5). However, the fluorescence of eGFP expressed from the few vectors exhibited variance in different strains, such as P*_10935_* (P8), which displayed strong promoter activity in all tested strains but not in *S. aureus* RN4220 (Figure 5). Importantly, most of the vectors expressed eGFPs with the same intensity in all treated *S. aureus* strains, which suggested that the promoters screened from *S. aureus* USA300 were functional in the same manner as those in other strains. These results indicated that the promoters we selected from *S. aureus* USA300 are conserved in *S. aureus* species, and the overexpression vectors constructed based on the identified active promoters were functional in different *S. aureus* strains with slight variance in their activity.

### Evaluation and Application of Developed Expression Vectors to Endogenous Gene Expression in *S. aureus*

**FIGURE 6:**
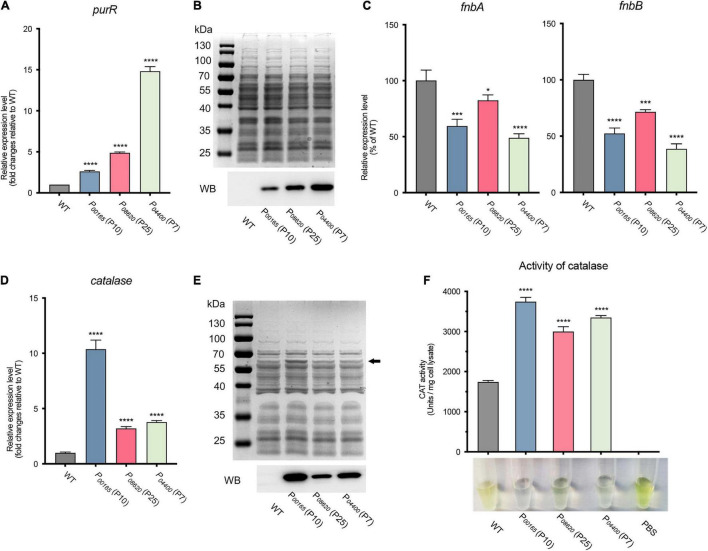
Evaluation and application of the developed expression vectors on endogenous gene expression in *S. aureus*. **(A)** The expression level of the *purR* gene in three overexpression vectors was validated by RT-qPCR. **(B)** The production of the PurR protein was evaluated by SDS-PAGE and western blotting. **(C)** The expression levels of the *fnbA* and *fnbB* genes, the target genes repressed by the transcriptional repressor PurR, were assessed by RT-qPCR in three *purR* overexpression and WT strains. **(D)** The expression level of the *catalase* gene in three overexpression vectors was assessed by RT-qPCR. **(E)** The production of PurR protein was evaluated by SDS-PAGE and western blotting. **(F)** The catalase activity of cell lysates from each overexpression strain and WT was measured by H_2_O_2_ and ammonium molybdate-based assays. The undecomposed hydrogen peroxide reacts with ammonium molybdate to produce a yellowish color. More transparency in the reacted mixture represents less hydrogen peroxide left, indicating higher catalase activity. Data shown are the mean ± SD of three experiments. (****P ≤ 0.0001, ***P ≤ 0.005; *P ≤ 0.05 relative to the WT).

To further evaluate and apply our constructed overexpression system, the endogenous gene *purR*, which encodes a transcriptional repressor, was expressed and validated in terms of its function in *S. aureus*. The PurR protein has been proven to be a transcriptional repressor of purine biosynthesis that inhibits the transcription of fibronectin-binding protein-coding genes *fnbA* and *fnbB* (Goncheva et al., 2019). Three overexpression vectors carrying promoters P*_00165_* (P10), P*_08620_* (P25), and P*_04400_* (P7) were randomly selected for overexpression of the *purR* gene. RT-qPCR analysis revealed that the *purR* gene was overexpressed in the three vectors compared to that in *S. aureus* WT (Figure 6A). The production of PurR protein (31.7 kDa) from strains overexpressing the *purR* gene with the P*_00165_* (P10), P*_08620_* (P25), or P*_04400_* (P7) promoter was further confirmed by SDS-PAGE and western blotting (Figure 6B). The signal detected from western blotting coincided with the transcriptional level of the *purR* gene as quantified by RT-qPCR, with the vector harboring P*_04400_* (P7) having the highest output, followed by P*_08620_* (P25) and P*_00165_* (P10). In addition, the physiological function of the overexpressed PurR protein was verified by checking the transcriptional level of its target genes *fnbA* and *fnbB* (Figure 6C). As shown, transcription of the *fnbA* and *fnbB* genes was significantly repressed in PurR overexpression strains compared to the WT (Figure 6C).

Furthermore, the endogenous catalase of *S. aureus* was overexpressed to further evaluate the constructed vectors. Similarly, overexpression of catalase using three vectors under the control of promoters P*_00165_* (P10), P*_08620_* (P25), and P*_04400_* (P7) was confirmed by RT-qPCR and western blotting (Figures 6D,E). The results showed that the transcription of the *catalase* gene in the overexpression strains was 3- to 10-fold higher than that in the WT, in which P*_00165_* (P10) possessed the highest transcription level, followed by P*_04400_* (P7) and P*_08620_* (P25) (Figure 6D). The same expression patterns of catalase were observed and confirmed by SDS-PAGE and western blotting (Figure 6E). In addition, the activity of catalase overproduced from different vectors was measured by H_2_O_2_ and ammonium molybdate-based assays. The undecomposed H_2_O_2_ reacts with ammonium molybdate to produce a yellowish color, i.e., more transparency of the reacted mixture represents less hydrogen peroxide left, which indicates higher catalase activity. The cell lysates from the overexpression strains exhibited significantly stronger catalase activity than those from the WT strain (Figure 6F). In line with the expression level, the vector carrying P*_00165_* (P10) that possessed the highest expression exhibited the highest catalase activity (Figure 6F).

In conclusion, the endogenous genes *purR* and *catalase* were successfully overexpressed by using our constructed overexpression vectors, and their related functions and phenotypes were confirmed in *S. aureus* USA300.

## Discussion

Currently, there are only a few genetic tools available for constitutive gene expression in *S. aureus*, which is largely due to the lack of characterized strong and reliable constitutive promoters. Here, we screened and evaluated 20 strong constitutive promoters for gene expression at a wide range of transcriptional levels in *S. aureus*.

A decent promoter used for constitutive gene overexpression principally possesses the characteristics of strong promoter activity in host cells. Thus, the top 1.5% highly expressed genes (RPKM ≥ 1,500) identified via RNA-seq in *S. aureus* were selected as the source of candidate promoter selection. Considering that the transcriptional level of a gene probably significantly varies during different growth phases (Mader et al., 2016), highly expressed genes in the lag phase, exponential phase, and stationary phases were filtered for promoter screening.

Since the exact -35 and -10 regions of the promoter from these genes are unknown, the gap non-coding sequence between the candidate gene and its upstream gene was defined as the potential promoter sequence. After the selected promoter sequences were cloned into a promoterless vector upstream of the beta-galactosidase reporter gene, the activity of these promoters was evaluated by measuring the beta-galactosidase activity of a strain transformed with the individual plasmid. As expected, not all selected promoters exhibited activity in the beta-galactosidase assay. This observation is presumably due to the incorrect selection of the promoter region, which is probably located within the coding region of the upstream gene, or the upstream gene utilizing the same promoter. Therefore, further work could be performed to identify the transcriptional start site of each highly expressed gene so that we can determine the exact -35 and -10 positions of these promoters. However, two-thirds of our cloned promoter sequences are active in *S. aureus*, which provides great potential for the development of a constitutive gene overexpression system.

Using the selected active promoters, a set of vectors was constructed for gene expression in *S. aureus*. All these generated vectors here are derived from the plasmid pBUS1_P*_*cap*_*_HC a *S. aureus*-*E. coli* shuttle vector constructed for gene expression under the control of the constitutive promoter P*_*cap*_* (Schwendener and Perreten, 2015). This vector is a promising backbone source for developing gene expression systems due to its characteristics of high copy number and high segregational stability in *S. aureus* (Schwendener and Perreten, 2015). Additionally, an RGS-6 × His coding sequence downstream of MCS enables target gene expression with a C-terminal polyhistidine-tag, allowing it to be directly detected or purified directly from *S. aureus*. Therefore, our developed overexpression vectors were constructed by replacing the P*_*cap*_* promoter sequence on pBUS1_P*_*cap*_*_HC with our identified promoters individually while keeping the other elements the same as the original.

After the expression vectors were constructed, the promoter strength or the ability of these vectors to express protein expression was evaluated and quantified by expressing an e*gfp* reporter gene (Figure 4). Unexpectedly, the promoter activity evaluated by eGFP fluorescence did not all correspond to that from RNA-seq. For example, P*_04400_* (P7) had lower activity than P*_01490_* (P3) in RNA-seq (Table 1), while P*_01490_* (P7) had higher promoter activity than P*_01490_* (P3) in the eGFP fluorescence assay (Figure 4). This observation was probably because some regulatory regions of the promoter were not included in our selected promoter sequences, which resulted in increasing or decreasing the strength of the promoter. In addition, eGFP expression does not always correlate with its transcript level, which could probably be caused by posttranscriptional regulation. The mRNAs of the *egfp* gene transcribed from different promoters have a different 5′ untranslated region (5′UTR). Some of the 5′ UTR regions contain regulatory elements that negatively or positively affect translation efficiency. Therefore, mRNAs with the same abundances might be translated to different amounts of proteins due to their different 5′ UTRs. In addition, it is worth noting that all these vectors were tested in *S. aureus* under lab growth conditions in TSB medium. If these are used in any other conditions, such as growing *S. aureus* in the presence of antibiotics or in a different medium, it would be better to evaluate these vectors first because the strength of a promoter may vary in different conditions (Mader et al., 2016). Nevertheless, our developed vectors with a wide range of promoter activity make it possible to constitutively express a certain protein at a desired level.

Because the promoters were screened from the strain *S. aureus* USA300, the universality of the vectors was evaluated by expressing the *egfp* gene in other clinical or laboratory strains. The expression levels of the *egfp* gene from a few vectors varied among different strains (Figure 5), which was probably caused by the slight differences in the genome sequence among each strain. For example, *S. aureus* RN4220 harbors many mutations in the genes encoding numerous regulators compared to *S. aureus* NCTC_8325 (Nair et al., 2011), which largely affects the transcriptional efficiency of their target promoters. However, most of the promoters exhibit the same strength as that of the native host *S. aureus* USA300, suggesting that our developed system is suitable for gene expression in different *S. aureus* strains. In addition, two endogenous genes, *purR* and *catalase*, were successfully expressed with our constructed vectors. The biological functions of the overexpressed proteins PurR and catalase were verified in *S. aureus* (Goncheva et al., 2019), which provides two practical examples for characterizing the function of unknown genes in *S. aureus* by using our developed overexpression system.

In the present study, we identified 20 strong constitutive promoters in *S. aureus* that exhibit a wide range of promoter strengths. Based on these promoters, a set of vectors for gene expression were constructed and evaluated. This system provides many choices for constitutive gene expression at different levels, thereby resolving previously limited genetic tools for gene expression. We believe that the expression system developed here will promote more in-depth studies on virulence genes or the function of unknown genes in *S. aureus*.

## Materials and Methods

### Bacterial Strains and Growth Conditions

The bacterial strains and plasmids used in this study are described and listed in Table 2. *E. coli* strains were grown in Luria-Bertani (LB) broth with constant shaking at 220 rpm or on LB agar plates at 37°C. *S. aureus* strains were cultured in tryptic soy broth (TSB) with shaking at 220 rpm or on TSB agar plates (TSA) at 37°C. Plasmids used for *S. aureus* transformation were modified by *S. aureus* RN4220. All *S. aureus* transformants were obtained through electroporation as described previously (Schenk and Laddaga, 1992). *E. coli* and *S. aureus* transformants were selected on agar plates containing 10 μg/mL tetracycline or 25 μg/mL chloramphenicol, and antibiotics were also used to maintain the plasmids in the cells.

### RNA Isolation and RNA-Seq

The day culture of *S. aureus* USA300 was prepared by inoculating 100 μL of an overnight culture into 10 mL of TSB medium in a 100-mL flask. Two milliliters of bacterial cells were collected after 2, 6, and 10 h of cultivation by centrifugation at 5,000 *g* for 10 min at 4°C. Bacterial cells were resuspended in 100 μL RNase-free cell lysis buffer (20 mM Tris-Cl, pH 8.0; 2 mM sodium EDTA; 1.2% Triton X-100; 50 μg/mL lysostaphin) and incubated at 37°C for 15 min. Then, RNA extraction was performed using an RNApure Bacteria Kit (CwBIO, Jiangsu, China) following the manufacturer’s instructions. The rRNA of the RNA samples was removed with a Ribo-Zero rRNA Removal Kit (Gram-positive Bacteria, Illumina) according to the manufacturer’s instructions. cDNA library preparation and sequencing were performed by Personalbio Co. (Shanghai, China).

### DNA Preparation and PCR Analysis

The isolation of genomic DNA and plasmid preparation using *E. coli* or *S. aureus* were performed with a bacterial genomic DNA kit and PurePlasmid Mini Kit, respectively (CwBIO, Jiangsu, China). Oligonucleotide primers were synthesized by Sangon Biotech (Shanghai, China). All primers used in this study are listed in Supplementary Table 2. For analytical purposes, PCRs were performed using OneTaq 2 × Master Mix (NEB, Ipswich, England). PCRs for plasmid construction were performed using Q5 High-Fidelity 2 × Master Mix from NEB according to the manufacturers’ instructions. The PCR products were purified using the DNA Clean-up Kit (CwBIO, Jiangsu, China). For cloning or plasmid construction, the plasmid was linearized by the related restriction enzymes from NEB. Cloning was performed using the ClonExpress II One Step Cloning Kit (Vazyme, Nanjing, China), which is based on homologous recombination.

### Construction of Vectors pQLV1001, pQLV1002, and pQLV1003

An entire chloramphenicol resistance cassette was amplified from plasmid pBT2 (Bruckner, 1997) using primers QL0230/0231 (Supplementary Table 2) and cloned into *S. aureus*–*E. coli* shuttle vectors pBUS1-HC and pBUS1-Pcap-HC (Schwendener and Perreten, 2015) at the *Bgl*II site, which generated vectors pQLV1001 and pQLV1002, respectively. To generate a promoterless beta-galactosidase reporter vector, a DNA fragment containing the red fluorescence protein gene (*rfp*) and beta-galactosidase gene (*lacZ*) was amplified from the pBS1C_*lacZ* plasmid (Popp et al., 2017) with primers QL409/0410 (Supplementary Table 2). The *rfp_lacZ* fragment was inserted into pQLV1001 linearized with *Hin*dIII and *Bam*HI to generate the *lacZ* promoter-probe vector pQLV1003.

### Promoter Cloning

The promoter regions of highly expressed genes (Supplementary Table 3) were amplified from the genomic DNA of *S. aureus* USA300 by PCR using the primers listed in Supplementary Table 2. Then, the promoter DNA fragment was individually cloned into the *lacZ* promoter-probe vector pQLV1003, which was linearized with *Eco*RI and *Pst*I. After confirmation by PCR and sequencing, the generated plasmid was modified by RN4220 and subsequently transformed into *S. aureus* USA300 for the beta-galactosidase-based promoter activity assay.

### Beta-Galactosidase-Based Promoter Activity Assay

The activity of the cloned promoters was first evaluated on the plate. Briefly, day cultures of *S. aureus* USA300 strains harboring the plasmid constructed above were prepared by inoculating 100 μL of the overnight culture into 10 mL of TSB medium. After the OD_600_ value reached 0.6, 5 μL of the bacterial culture was pipetted out and dropped onto a TSA agar plate containing 25 μg/mL chloramphenicol and 200 μg/mL *X*-Gal (5-bromo-4-chloro-3-indolyl-β-D-galactoside). The plate was incubated at 30°C, and the color of the colony was monitored and photographed after 48 h.

To further quantify the promoter activity, analysis of beta-galactosidase activity using *O*-nitrophenyl-β-D-galactopyranoside (ONPG) as substrate was performed and modified as previously described (Zhu et al., 2020). A single blue colony on the TSA *X*-Gal plate was inoculated into chloramphenicol-containing TSB medium overnight culture preparation. The next day, the day culture was prepared as described above. After 2, 6, or 10 h of cultivation, 100 μL of the bacterial culture was collected for OD_600_ value measurement in a 96-well plate by using a Synergy H1 microplate reader (Vermont, United States).

Meanwhile, bacterial cells were harvested from the 200 μL culture at each time point by centrifugation at 5,000 *g* for 10 min at 4°C. The cells were resuspended in 100 μL of lysis buffer (60 mM K_2_HPO_4_, 40 mM KH_2_PO_4_, 100 mM NaCl, 0.1% Triton X-100, 50 μg/mL lysostaphin) for total protein extraction. The suspension was incubated at 37°C until the bacterial cells had completely lysed. Then, 50 μL of the lysate was pipetted onto a 96-well plate for OD_420_ measurement. After that, 100 μL of a reaction buffer (60 mM K_2_HPO_4_, 40 mM KH_2_PO_4_, 100 mM NaCl, 0.1% Triton X-100, 5 mg/mL ONPG) was added to each well and mixed with the cell lysate. The 96-well plate was incubated in the Bioreader at 37°C with constant shaking, and the OD_420_ was measured every 5 min. The slope of the linear part of the spectrophotometric output was used to calculate the specific activity as follows: Miller units = (1,000 × slope)/(V × OD_600_), where V is the volume (in milliliters) of the culture used in the assay. This assay was repeated three times for each strain.

### Construction of Overexpression Vectors and Their Derived Plasmids

The promoter sequences of the validated active sequences (Supplementary Table 3) were amplified by PCR from the pQLV1003-derived plasmids carrying different promoters using the primers listed in Supplementary Table 2. A fragment “AGGAGGTTTAT*CATATG*” that contained a typical RBS (underlined), a spacer, and an *Nde*I site (italic) was introduced downstream of each promoter from the primer. This will lead the target gene to be cloned at the *Nde*I site and other restriction enzyme sites downstream of *Nde*I. The amplified promoter was cloned into vectors pQLV1002 linearized with *Kpn*I and *Nde*I to generate a series of overexpression vectors.

To evaluate the constructed expression vectors, the *egfp* reporter gene was PCR amplified from plasmid pTH100 (de Jong et al., 2017) with primer pairs QL0558/0559 and inserted into each constructed expression vector that was linearized with *Nde*I and *Xho*I. The endogenous transcriptional repressor *purR* or *catalase* gene of *S. aureus* USA300 was PCR amplified with the primer pairs QL0609/0610 and QL0613/0614, respectively. These were cloned into the overexpression vectors at the *Nde*I and *Xho*I sites for PurR and catalase expression with an RGS_6 × His tag at their C-termini.

### Fluorescence-Based Promoter Activity Assay

A day-culture was prepared as described above. Bacterial cells from the 200 μL day-culture after 2, 6, and 10 h of inoculation were harvested by centrifugation. Cells were washed twice with 500 μL of PBS buffer and suspended in PBS to an OD_600_ value of approximately 0.3. The OD_600_ value and relative light unit (RLU) of GFP fluorescence (excitation, 485 nm; emission, 512 nm) were measured using a Synergy H1 plate reader. Promoter activity was analyzed by calculating the ratio of RLU/OD_600_. The assay for each strain was performed in three independent experiments, and the mean value of RLU/OD_600_ was used to generate the heatmap.

### Real-Time Quantitative PCR

The qPCR was performed in a CFX-96 Touch Real-Time PCR system (Bio-Rad, Hercules, CA, United States) using SYBR green master mix TB Green *Premix Ex Ta* II (Takara, Beijing, China). Total RNA from *S. aureus* was isolated as described above. Approximately 1 μg of total RNA was used for reverse transcription using a PrimeScript™ RT reagent kit with gDNA Eraser (Takara, Beijing, China) according to the manufacturer’s instructions. After the transcribed cDNAs were diluted fivefold, 2 μL of the cDNA was used as DNA template in 15-μL amplification volumes with 400 nM of each primer and 7.5 μL of SYBR green master mix using the following cycling parameters: 95°C 30 s; followed by 40 cycles of 5 s at 95°C, 30 s at 55°C, and 30 s at 72°C. The primer pairs QL0615/QL0616, QL0619/QL0620, QL0633/QL0634, QL0635/QL0636, QL0637/QL0638, and QL0645/QL0646 were used to amplify the e*gfp*, *purR*, *catalase*, *fnbA*, *fnbB*, and *sigA* genes, respectively, by qPCR. The endogenous gene *sigA*, which encodes the housekeeping RNA polymerase sigma factor, was used as an internal control for promoter characterization. The expression levels of the *egfp* gene under different promoters were normalized to the expression of the internal control.

### Western Blot Analysis

Total protein extracts of *S. aureus* were prepared as described in the beta-galactosidase assay. The protein concentration was measured using a Bradford assay kit (Thermo Scientific, Waltham, MA, United States). Twenty micrograms of the total protein extract was separated by 12% SDS-PAGE and transferred onto a nitrocellulose membrane (Bio-Rad). The membrane was blocked with TBS-T buffer (20 mM Tris-HCl pH 7.5, 150 mM NaCl, 0.1% [vol/vol] Tween-20) containing 5% non-fat dry milk at room temperature (RT) for 1 h. Detection of the target proteins eGFP_6 × His, PurR_6 × His, and catalase_6 × His overexpressed in *S. aureus* was performed by incubating the membrane with the horseradish peroxidase (HRP)-labeled His-Tag (27E8) mouse mAb (CST, Danvers, MA, United States) (diluted 1:2,000 in TBS-T buffer) at RT for 1 h. After four washing steps (10 min per step) with TBS-T buffer, the signals were detected with a ChemiDoc MP (Bio-Rad) imaging system using Pierce ECL (Thermo Scientific, Waltham, MA, United States) as a chemiluminescence substrate.

### Catalase Activity Assay

Bacterial cells from the 200 μL day-culture were collected in the middle of the lag growth phase (6 h after inoculation) by centrifugation. The precipitated cells were resuspended and lysed in 800 μL of phosphate-buffered saline (PBS, pH 7.4) by sonication on a SCIENTZ-IID system (SCIENTZ, Ningbo, China) according to the user guide. The protein concentration of the cell lysate was measured as described above. To measure the activity of catalase in the lysate, 20 μL of the cell lysate was mixed with 100 μL of 200 mM H_2_O_2_ (diluted with PBS) and incubated at 25°C for 10 min. Then, the reaction was stopped by adding 180 μL of ammonium molybdate (50 mM in H_2_O) into the mixture. After incubation at room temperature for 10 min, the absorbance of the yellow complex of molybdate and undecomposed hydrogen peroxide was measured at a wavelength of 405 nm in a 96-well plate (*A*_Sample_) using the plate reader Synergy H1 (BioTek, Vermont, VT, United States). One unit of catalase activity was defined as 1 μM of H_2_O_2_ catalyzed and hydrolyzed by 1 mg lysate in 1 min at 25°C. Thereby, the catalase activity of the lysate (CAT) was calculated as follows: CAT (U/mg) = [(Δ*A*_Standard_ − Δ*A*_Sample_)/Δ*A*_Standard_] × N/m/T × F, where Δ*A*_Standard_ = *A*_Standard_ − *A*_Blank_ and Δ*A*_Sample_ = *A*_Sample_ − *A*_Control_. *A*_Control_ is the absorbance of the control group, in which H_2_O_2_ was replaced by PBS. A_Standard_ is the absorbance of the standard group, in which the cell lysate was replaced by PBS, while *A*_Blank_ is the absorbance of the blank group, in which cell lysate and H_2_O_2_ were both replaced by PBS. *N* represents the molar mass of H_2_O_2_ (micromole) used in the assay, and m is the mass of the cell lysate (mg) used in the assay. T is the reaction time in minutes, and F is the dilution factor of the cell lysate. The assay for each strain was performed in three independent experiments.

## Data Availability Statement

The datasets presented in this study can be found in online repositories. The names of the repository/repositories and accession number(s) can be found in the article/Supplementary Material.

## Author Contributions

QL: funding acquisition, project administration, investigation, methodology, writing, review, and editing. DL, NW, GG, YS, and QZ: data curation, formal analysis, investigation, methodology, visualization, writing original draft, and editing. XZ: project investigation, administration, writing, review, and editing. All authors contributed to the article and approved the submitted version.

## Conflict of Interest

The authors declare that the research was conducted in the absence of any commercial or financial relationships that could be construed as a potential conflict of interest.

## Publisher’s Note

All claims expressed in this article are solely those of the authors and do not necessarily represent those of their affiliated organizations, or those of the publisher, the editors and the reviewers. Any product that may be evaluated in this article, or claim that may be made by its manufacturer, is not guaranteed or endorsed by the publisher.
